# Applications of Ion Mobility-Mass Spectrometry in Carbohydrate Chemistry and Glycobiology

**DOI:** 10.3390/molecules23102557

**Published:** 2018-10-07

**Authors:** Yuqing Mu, Benjamin L. Schulz, Vito Ferro

**Affiliations:** 1 School of Chemistry and Molecular Biosciences, The University of Queensland, Brisbane 4072, Australia; yuqingmu0708@hotmail.com (Y.M.); b.schulz@uq.edu.au (B.L.S.); 2 Australian Infectious Diseases Research Centre, The University of Queensland, Brisbane 4072, Australia; 3 Australian Research Council Industrial Transformation Training Centre for Biopharmaceutical Innovation, The University of Queensland, Brisbane 4072, Australia

**Keywords:** carbohydrates, isomeric ions, ion mobility-mass spectrometry (IM-MS)

## Abstract

Carbohydrate analyses are often challenging due to the structural complexity of these molecules, as well as the lack of suitable analytical tools for distinguishing the vast number of possible isomers. The coupled technique, ion mobility-mass spectrometry (IM-MS), has been in use for two decades for the analysis of complex biomolecules, and in recent years it has emerged as a powerful technique for the analysis of carbohydrates. For carbohydrates, most studies have focused on the separation and characterization of isomers in biological samples. IM-MS is capable of separating isomeric ions by drift time, and further characterizing them by mass analysis. Applications of IM-MS in carbohydrate analysis are extremely useful and important for understanding many biological mechanisms and for the determination of disease states, although efforts are still needed for higher sensitivity and resolution.

## 1. Introduction

Mass spectrometry (MS) is the most widely used technique for glycan analysis. Coupled with gas chromatography (GC) or liquid chromatography (LC) as pre-separation tools, MS is capable of separating isomeric ions based on retention times. However, glycan analyses by MS are challenging due to the structural complexity of glycans and the inability of MS to distinguish isobaric structures. Ion mobility spectrometry (IMS) is a technique utilized for isomeric ion separation prior to mass analysis which is characterized by rapid separation times, high resolving power and high reproducibility [1,2]. The coupling of IMS with MS (IM-MS) has developed rapidly into a powerful and widely used separation technique with applications in many areas across the biological sciences, including the glycocsciences. With the first application of IM-MS for distinguishing oligosaccharide isomers in 1997 and the first application of IM-MS for separating and elucidating isomeric carbohydrates from mixtures in 2005, the application of IM-MS in the glycosciences [3,4] has seen a rapid growth in the past decade, as exemplified by several recent reviews [1,5,6,7,8,9]. In this contribution, we provide an overview of IMS and IM-MS, including a discussion of the different types of instrumentation. We then focus on the applications of IM-MS in the glycosciences from the 1990s to mid-2018.

## 2. Complexity and Standard Analytical Techniques for Carbohydrates

### 2.1. Complexity of Carbohydrates

Carbohydrates, found both intracellularly and on the cell surface in the form of glycoconjugates, are extremely important biomolecules for all living things [10]. They participate in many biological processes including cell-to-cell recognition and signaling, protein folding, receptor binding, embryonic development and differentiation, and immune responses [4,11,12]. Therefore, understanding their structures, functions and interactions with other biomolecules in as much detail as possible, is of great interest. However, a major problem for studying carbohydrates is the complexity of their structures and the limitations of the available analytical tools.

**Figure 1:**
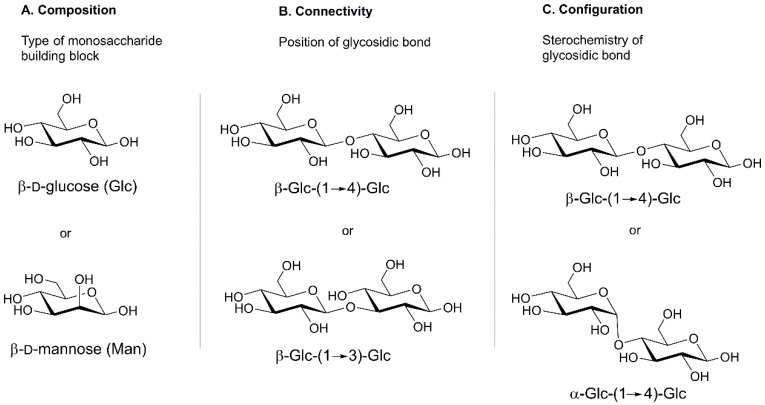
The complexity of carbohydrate structures results from the constitution and spatial distribution of monosaccharides as well as the connectivity and configuration of the glycosidic bonds that link sugar molecules. Adapted from Hofmann et al. [14].

The structures of carbohydrate are extremely complex due to the existence of numerous isomeric forms which result from various positional isomers, linkage types (α or β) and configurations of various monosaccharide building blocks (see Figure 1) [11,13,14,15]. Considering the diversity of structures of simple saccharides, it is not surprising that the difficulty in studying carbohydrates increases with more complex structures (e.g., glycans, glycopeptides, and glycoproteins), that contain multiple distinctly or non-distinctly different monosaccharide units with the presence of biomolecules from different biological classes.

### 2.2. Standard Analytical Techniques for Carbohydrates

Mass spectrometry has been extensively adopted for carbohydrate structural analysis [2]. Although carbohydrate structural information obtained by MS is usually impeded by the complexity of isomeric precursor mixtures, the addition of gas chromatography (GC) or liquid chromatography (LC) prior to mass analysis can reduce the complexity of isomeric ion mixtures by separating ions by retention times, and facilitate the structural identification of saccharides [2]. In fact, the combination of LC and tandem MS is regarded as the “gold standard” for glycan analysis [1]. However, the current gold standard is still less than ideal for glycan analysis because: (a) it has limited sample throughput, (b) it cannot distinguish ions with the same mass and retention time, and (c) it is not rapid enough [16,17,18].

## 3. Overview of Ion Mobility-Mass Spectrometry

### 3.1. Introduction of IM-MS

Ion mobility-mass spectrometry (IM-MS) is a versatile two-dimensional analytical technique for rapid separation and simultaneous detection of compounds of interest by conducting a gas-phase separation prior to mass analysis. IM-MS developed rapidly into a ready-to-use technique as it became commercially available from 2006, and its usefulness in various fields has become apparent, especially for glycan analysis [5,19]. The earliest literature regarding the IM-MS technique appeared in the early 1960s [20,21,22]. It was subsequently greatly utilized in a variety of applications including biological [22,23], clinical and therapeutics [17,24,25], pharmaceuticals [26,27,28,29], explosives [30] and chemical warfare agents [31]. The biological applications of IM-MS cover oligonucleotides, carbohydrates, peptides, proteins and lipids, that are derived from various sources, e.g., bovine submaxillary mucin (BSM), human cholesterol, human blood samples, urine samples from patients, glycoproteins and ovalbumins, etc. [4,16,17,24,25,27,32,33,34,35,36]. IM-MS is more extensively used in biological areas than several other traditional, widely used techniques because of the limitations of these other techniques. For example, immunoassays cannot analyse biological samples with complex structures due to the presence of matrix effects and possible cross reactions [33]; capillary electrophoresis (CE), as a separation tool is often incompatible with the commonly used ionization source, electrospray ionization (ESI), since it detects chemically modified molecules in solution conditions [37]; LC fails to distinguish isomers that migrate together [32]; NMR is not powerful enough to provide data for structural elucidation for biological samples that are mixtures of isomers due to its high sample requirements [6,15,32]. In addition, the peak capacity of MS is increased by a magnitude of six when coupled with IMS which contributes to the enhancement of resolving power [17]. IM-MS also has advantages over some traditional techniques used in combination for biological analysis. Specifically, MS/MS and MSn cannot separate precursor ions that give rise to product ions with the same mass-to-charge ratio (m/z) [32,36]; GC-MS requires derivatization of samples of interest [33]; LC-MS has a more than three-fold slower peak production rate than IM-MS [16,17] and HPLC-MS cannot recognize the existence of some isomers in mixtures [32,38]. The additional resolution power offered by IMS is therefore critical to its performance in separation of carbohydrates.

Before coupling with MS, IMS was simply utilized for separating individual isomeric ions [4] while MS was sometimes coupled with ESI, GC, LC or HPLC to analyse complex samples of interest [32,33,39]. In 1997, individual oligosaccharide isomers were distinguished by injected ion mobility/MS and it was the first application of ion mobility methods for carbohydrates [3]. In 2005, IM-MS was used to separate and elucidate isobaric carbohydrates from mixtures for the first time [4]. Before 2005, few studies regarded IM-MS as a combination to analyse samples from different sources [40,41]. With the emergence of the first commercially available IM-MS instrument in 2006 [19], it was then largely utilized in research for complex structures from complex mixtures in numerous areas.

In general, IM-MS was shown to be able to characterise conformers, chiral molecules and isomers even without prior knowledge of their structure [13,31], and to provide dissociation spectra of each. Its applications covered various fields from chemical analysis such as real-time nicotine monitoring [42] and detection of explosives [30], biological analysis of oligonucleotides, lipids, peptides, proteins and carbohydrates [17,22,23,34,43], to military and civil environs [31]. For chemical analysis, Eiceman replaced traditional detection techniques with the more rapid IM-MS in 1995, because IM-MS was capable of real-time, continuous detection of characteristic product ions and effectively recognizing and characterizing nicotine ion clusters, while the accumulation and preparation process of traditional methods were not fast enough to do so [42]. In 2001, Eiceman and colleagues reported the significance of IMS for detection of trace levels of nitro-organic explosives [30]. Later, IM-MS was applied to studies on isomeric di-halogenated benzenes and morphine levels in saliva [44,45]. With the popularity of IM-MS, more advantages were illustrated. Other than for its high speed, sensitivity and resolution, the simplicity of handling and the low lifetime cost were also key factors for its popularity [31].

Biological applications of IM-MS include simultaneous qualitative separation of biomolecules of different classes, examination and prediction of fine structures within a biological class with further computational strategies, such as molecular dynamics simulations [23,34]. For biomolecules of different classes, there is a decreasing order of average density as follows: oligonucleotides, carbohydrates, peptides and lipids [23,34]. So, with a given m/z, the volume as well as drift time of these biomolecules would decrease in the order: lipids, peptides, carbohydrates and oligonucleotides. According to the theoretical relationship between the scale of drift time and relative collisional cross section (CCS) with specific separation conditions (see Equations (1)–(5)), the slopes for biomolecules from different classes would be distinctive by plotting drift time (CCS)-m/z relationship in a coordinate system [22,23]. So it is possible to predict the relative CCSs of biomolecules if we know which biological category it falls into. Additionally, structural information of biomolecules is also obtainable through comparing molecular dynamics modelling with CCS values derived empirically with specific separation conditions [34].

For peptides, IM-MS could not only separate conformational and isomeric ions directly from samples but it also revealed promising primary structural information [40,46,47,48,49], tracked structural changes by coupling with collision induced unfolding (CIU) experiments [50], and allowed the building of plasma proteome maps [51]. For metabolites, IM-MS was utilized more exclusively than any other traditional technique due to the existence of chemical diversity, isobars and chiral compounds in metabolites, an extremely wide range of physiological concentrations of metabolite samples, as well as low sensitivity of NMR at low concentrations, chemical non-selectivity of IR, insensitivity of GC for non-volatile compounds without derivatization, relatively low speed and resolution of HPLC, and relatively high chemical noise of ESI-MS [52]. Studies regarding the adoption of IM-MS have been successfully conducted in E. coli metabolites [52,53], human blood metabolites [17], and drug metabolites [29]. The application of IM-MS for metabolites facilitates metabolite database building and novel compound characterization and diagnosis [52,53], disease progression prediction [17] and pharmacology of drugs [29]. For hormones, IM-MS was more preferable than other analytical methods because immunoassays had matrix effects as well as possible cross reactions, GC-MS was time-consuming and LC-MS was less effective on less polar steroids [33]. Studies have been conducted for separating hormone steroids [33], studying their formation and stabilities of conformations [54]. For lipids, IM-MS improved the analysis which was impeded by the existence of isomer interferences and detection suppression of low concentrations by those with high abundance and endogenous and exogenous chemical noise [34,55].

### 3.2. Principles of IM-MS

MS is an essential analytical tool for analyte identification based on mass-to-charge ratios (*m*/*z*). It is utilized extensively in chemistry and biology because it allows rapid analysis of complex samples on a microsecond scale with high sensitivity and requires only small sample volumes [38]. Despite its high sensitivity, MS still has limitations. Specifically, MS can neither give straightforward information on three-dimensional structures of samples of interest nor separate fractions with similar *m*/*z*, isobars nor species that serve as contributors to chemical noise [11,18]. In order to elevate the sensitivity of MS and expand its applications, efforts have been made to acquire MS/MS spectra and MS*n* spectra [38], or to combine MS with several other traditional separation techniques, including GC, LC and high performance liquid chromatography (HPLC), and most recently, IMS [18,34,38,55].

IMS, also called plasma chromatography and ion chromatography, is a high throughout separation technique based on fine structures of compounds of interest, and it is not a newly emerged technique [18,23,34]. Its concept of separating gas ions by migration time through a neutral buffer gas with an applied field has existed for over a century [23]. It was firstly used for atomic and molecular ion studies before it was introduced as a separation tool in analytical and physical chemistry in the 1960s and applied to biological research in the mid-1990s for structural analysis of peptides and proteins [23,46,48]. Specifically, it separates ionized gas samples by ion mobility, which can be characterized through arrival time or drift time and determined by ion size, shape, mass and charge [16]. Drift time is the amount of time that a gas ion spends to flow against neutral gas, e.g., nitrogen or helium, in a long drift tube applied with static electricity. Degrees of collision between ionized gas and neutral gas due to the diversity of sizes and shapes of gas ions in analytes, can be calculated directly through drift time. After combining with MS spectra, it can be integrated into databases for future structural identification [56]. Compared with gas chromatography (GC) and liquid chromatography (LC) which both serve as separation tools, IMS separations require nearly 4~5 orders of magnitude less time than GC or LC [18]. Therefore, due to its rapidity and reproducibility, IMS is advantageous for analysis of ionic samples that are transiently isolated [2].

IM-MS separates gas-phase ion samples in an electric field through a long drift tube prior to mass analysis on independent fragmentations of isomeric ions [16]. Gas ions flow through the drift tube under the joint action of electrostatic force and resistance to reversed neutral gas flow before they are trapped for mass analysis. Normally, higher charged ions have shorter drift times than lower charged ones because electrostatic force is proportional to charge state. Ions whose structures are more compact have shorter drift times than stretched ones due to smaller areas on their surfaces that collide with buffer gas. As mentioned above, drift time can be utilized as a reproducible transport property to underpin structural databases when converted into CCS after standardized calculations. CCS reveals underlying structural information on the surface of gas ions compared with theoretical CCS, a size parameter related to the surface of molecules obtained from molecular modelling, for an empirical deduction of shape and size of each fraction of gas ion [23,29,57,58]. IM-MS employs two orthogonal types of separation to obtain mass-mobility spectra with mobility peaks and mass analysis of each mobility peak on a millisecond timescale. By combining two-dimensional mass-mobility spectra with one-dimensional mobility profiles, with a certain *m*/*z* value, one can obtain straightforward information on the presence of isomers through the number of mobility peaks because each mobility peak represents an isomeric form [13,32]. By combining two-dimensional mass-mobility spectra with anomer concentrations, one can get straightforward information on the relative content of anomers in crude sample mixtures even at an extremely low concentration (0.1%) [14].

### 3.3. IM-MS Instrumentation

**Figure 2:**
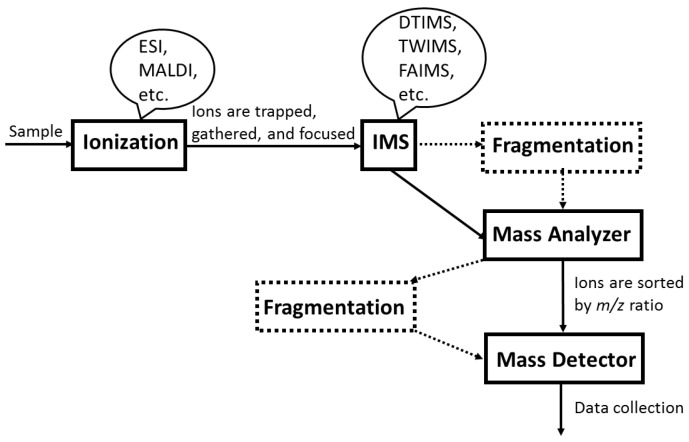
A simplified model for a typical IM-MS instrument.

A simplified illustration of an IM-MS instrument is shown in Figure 2. Gas-phase samples are ionized by ESI or MALDI. Ionized molecules are trapped, gathered, and focused through funnels before entering the ion mobility spectrometer, such as DTIMS, TWIMS and FAIMS (*vide infra*). Ions flow through a long drift tube in the spectrometer and are separated either in a time-dispersive or space-dispersive manner. After refocusing, ions are sorted and analysed in the mass spectrometer. Extra fragmentation tools could be utilized either prior to or after mass analysis.

**Figure 3:**
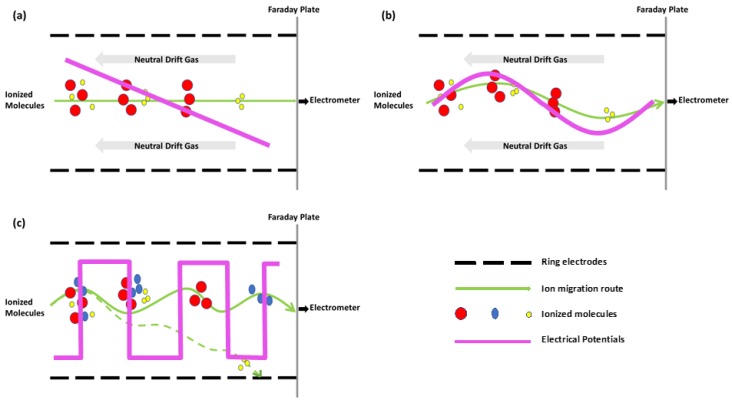
An illustration of the drift area of different IMS instruments. (**a**) DTIMS: ionized molecules travel against neutral drift gas through a uniform electrostatic field and are separated in a time-dispersive manner; (**b**) TWIMS: ionized molecules travel against neutral drift gas through an electrodynamic field and are separated in a time-dispersive manner; (**c**) FAIMS: ionized molecules travel flow through an electric field applied with greatly varied voltage and are separated in a space-dispersive manner such that only ions with specific mobility reach the electrometer.

In general, an IM-MS instrument consists of an ion mobility drift tube with an ionization source followed by a mass spectrometer. The most commonly used ionization sources are electrospray ionization (ESI) and matrix-assisted laser desorption/ionization (MALDI) [34,46]. Atmospheric pressure chemical ionization (APCI) also appeared to produce stable product ions in a few early papers prior to the emergence of ESI and MALDI [30,42]. The main difference between ESI and MALDI is that ESI ionizes samples from solution while MALDI ionizes samples directly from a solid matrix [43]. While choosing a suitable ionization source, one must take the following into consideration: (a) normally, ESI produces singly charged ions for small biomolecules and multiply charged ions for large biomolecules while MALDI only produces singly charged ones [43]; (b) MALDI requires extra empirical selection of matrices but is more resistant to salt than ESI [43]; (c) ESI produces ions continuously while MALDI produces ions in pulse mode [43]. There are several kinds of IMS instruments, ambient pressure ion mobility spectrometer (APIMS), field asymmetric waveform ion mobility spectrometer (FAIMS), drift-tube ion mobility spectrometer (DTIMS), traveling-wave ion mobility spectrometer (TWIMS) or the combination of TWIMS and DTIMS (see Figure 2 and Figure 3) [31,57]. There are also several kinds of MS instruments, time-of-flight mass spectrometer (TOFMS), quadrupole mass spectrometer (Q-MS), triple quadrupole mass spectrometer, quadrupole ion trap mass spectrometer (QITMS), Fourier transform ion cyclotron resonance mass spectrometer (FTICR) and sometimes the combination of QITMS and TOFMS, also known as QIT-TOFMS [13,32,57]. Before ESI, IMS and MS were used in combination, many reports indicated the utilization of ESI and MS in combination [11,26,33,52,56]. The addition of IMS, an extremely rapid separation step which does no harm to the high resolution and throughput of tandem MS, not only reduces baseline noise but also facilitates the separation of isobaric ions in complex mixtures [52]. Most recently, with a 5-fold higher resolution of separations than commercially available DTIMS or TWIMS, the higher resolution platform of IMS, Structures for Lossless Ion Manipulations (SLIM) platform, has been able to resolve glycoconjugates with a single stereochemical difference within a monosaccharide [59]. The ultra-high resolution of SLIM IM-MS enabled it to distinguish lipids and glycolipids with slightly different structures [60].

### 3.4. Limitations of IM-MS

Although IM-MS is an extremely rapid separation technique with high throughput and high sensitivity for analysing complex samples from different sources, it still has some limitations. Specifically, overlap may still occur between isobars and non-isobaric species with similar drift time. This may cause inaccurate CCS calculations and incorrect identification of sample structures even with tandem separation or post-IMS identification techniques [61]. The limitations of IM-MS have been discussed in many publications. For example, (a) IM-MS is unable to separate multiple coexistent isomers because multiple cross-sectional areas and some isomeric forms in the structures might migrate together [2,19], such as in large oligosaccharides and large isomeric glycans [32,56]; (b) IM-MS is not powerful enough for biomolecules with relatively flexible structures due to the lack of convincing protocols for CCS calculations and suitable molecular modelling techniques [29,58]. Several studies also illustrated possible solutions to improve these limitations of IM-MS including: (a) running analytes through a compatible separation instrument, such as LC or HPLC, prior to IM-MS for a better supplement of CCS information [19,22,23]; (b) coupling IM-MS with hydrophilic interaction liquid chromatography (HILIC), which is a pre-separation technique that utilizes the interaction between hydroxyl groups of polar analytes and polar groups on the stationary phase surfaces [56,62]; (c) combining existing IM-MS data with chemometric techniques, such as principle component analysis (PCA) and simple-to-use interactive self-modelling mixture analysis (SIMPLISMA) [61]; (d) utilizing hybridized (IM-MS)^2^ for a higher resolving power [22,63]; (e) suitable derivatization of samples of interest for higher selectivity in IM-MS [4,64]; (f) running samples through IM-MS with the additional collision-induced dissociation (CID), or vacuum ultraviolet photo-dissociation (VUVPD) prior to mass analysis or prior to IMS [37,65]; (g) coupling IM-MS with gas-phase IR spectroscopy for more detailed information analysing extremely similar structural isomers [8].

**Figure 4:**
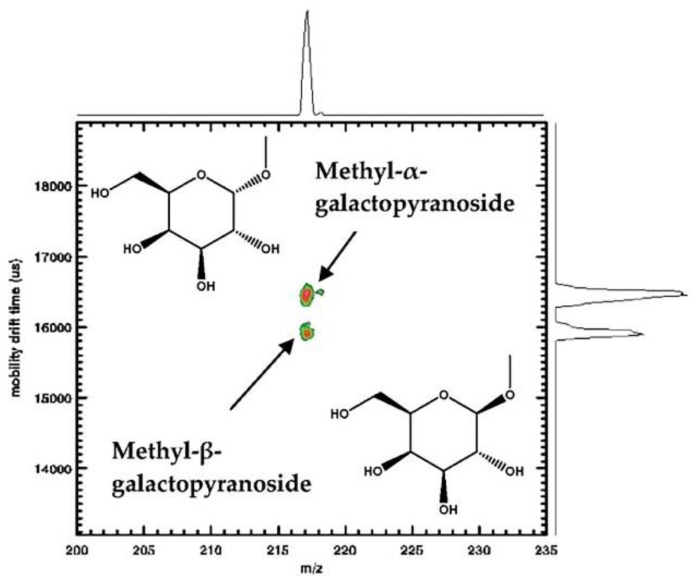
Unambiguous separation of two monosaccharide isomers by IM-MS [12] (reproduced with permission).

The rapidity, high resolution and selectivity of IMS as a separation tool is of great use considering the complexity and transiency of biomolecule structures. For carbohydrate studies, IM-MS has been shown to be a promising method [22,23,34,43]. IM-MS separates fragment ions by mobility and analyses the mass-to-charge ratio (*m*/*z*) of each fragmentation to obtain IM-MS spectra. IM-MS spectra are extremely useful to identify the existence of isomers and further distinguishing them with experimental data by combining two-dimensional mass-mobility correlation spectra with the additional one-dimensional mobility profiles for selected *m*/*z* components in different fractions from different biological resources (see Figure 4) [4,12,32]. After combining mass-mobility correlation spectra with the corresponding mobility profiles, one can obtain mobility spectra (shown on the right-hand side), mass-mobility correlation spectra (in the center) and the mass peak of each biomolecule (on the top) of the spectrum (see Figure 4).

Figure 4 shows a single mass peak at *m*/*z* 217 and two partly separated mobility peaks on the right hand side, indicating these two components (methyl-α-d- and methyl-β-d-galactopyranoside) have identical *m*/*z* but differ in ion mobility. Based on their structures shown on the left hand side, the identical mass (*m*/*z* 217) was reflected as the single mass peak on the top of the spectrum while the different anomeric linkage resulted in the separation of drift time on the right hand side. Given isomers differ in structure, a significant determinant of drift time, instead of *m*/*z* values, it becomes reliable to define isomers by mobility peaks. With specific *m*/*z* values, mobility peaks represent isomeric forms [32].

In order to clarify which isomer is represented by a specific mobility peak, calculations are required. With the simplest IMS, DTIMS, the experimental mobility (*K*) and normalized mobility (*K*_0_), the ion-neutral collision cross section (*Ω*) of each ion, the resolving power (*R_p_*) of the technique and resolution (*R*) of the specific separation are straightforward to calculate [4].

The mobility (*K*) of an ion is defined as the quotient of drift velocity (*v_d_*) and the uniform electric field (*E*). *v_d_* is the ratio of total length of drift tube (*L*) and drift time (*t_d_*) that the ion spends to traverse the drift tube. *E* is obtained through the potential (*V*) applied to the entire drift area:

(1) $$K=\frac{\nu_{d}}{E}=\frac{L^{2}}{t_{d}V}$$

*K*_0_ is the standardized value of experimental *K*. Normalization of *K* values is aimed to make comparison among identical and distinctive ions under different experimental conditions regarding the impact of temperature (*T*) and pressure (*P*) of inert drift gas on experimental data obtained:

(2) $$K_{0}=K\frac{273.15}{T}\frac{P}{760}$$

The separation power (*R_p_*) illustrates the separation power of IM-MS and it is attainable through a division operation between *t_d_* and half-peak width (*W*_1/2_):

(3) $$R_{p}=\frac{t_{d}}{W_{\frac{1}{2}}}$$

*R* of the separation between two species is defined as the quotient of the difference in drift time (Δ*t_d_*) and the average baseline peak width ($\bar{W_{base}}$). It is used to interpret the degree to which mobility peaks are separated. The higher the *R* value is, the more completely two species are separated. Normally when the *R* value reaches 1.5, it can be regarded as baseline separation [4]:

(4) $$R=\frac{\Delta t_{d}}{{\bar{W}}_{base}}$$

The calculation of CCS (*Ω*) of an ion is shown below where every element is converted into the standardized value under standard experimental conditions (see Equation (5)). *N*, the number density of the inert drift gas; *μ*, the reduced mass of an ion (*m*) and a neutral inert drift gas (*M*): [*μ* = *mM*/(*m* + *M*)]; *k*, Boltzmann’s constant (*k* = 1.38 × 10^−23^ *J*/*K*); *ze*, charge state of an ion (*e* = 1.6 × 10^−19^ *C*).

(5) $$\Omega =\left(\frac{3}{16N}\right){\left(\frac{2\pi }{\mu kT}\right)}^{\frac{1}{2}}\left(\frac{ze}{K_{0}}\right)$$

CCS, as a parameter, is also valuable for the construction of databases of carbohydrates with distinctive structures, calibration of methods and the prediction of specific structures with known mass and mobility. The significance of CCSs in specific cases is included in the following sections.

## 4. Applications of IM-MS to Carbohydrates

### 4.1. Mono- and Oligosaccharides

**Figure 5:**
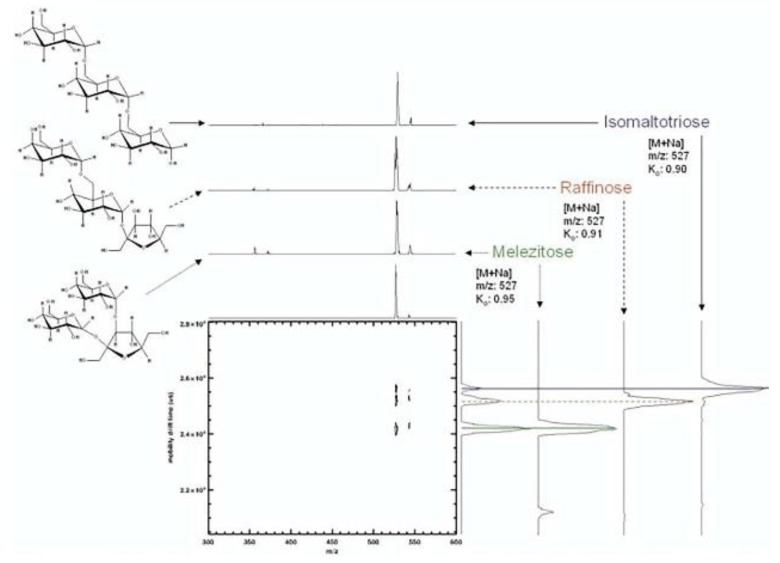
Structures and IM-MS spectra of a mixture of trisaccharide isomers melezitose, raffinose and isomaltotriose [4] (reproduced with permission).

The identification of oligosaccharides in biological samples has long been impeded by the failure of rapid differentiation of isomeric forms and evaluation of isomeric heterogeneity in chromatographic or mass spectra [12]. The first report of unambiguous physical resolution of carbohydrate isomers from mixtures utilizing atmospheric pressure high resolution ion mobility spectrometry coupled with time-of-flight mass spectrometry (APIMS-TOFMS) was conducted by Hill and coworkers in 2005. They separated four sodium adducted disaccharides released from bovine submaxillary mucin (BSM) linked α1–3, α1–6, β1–3, or β1–6, and three isomeric sodium adducted trisaccharides, melezitose, raffinose and isomaltotriose by IM-MS to illustrate the separation ability of the technique. In their study, linkage isomers α1–3 and α1–6, β1–3 and β1–6 were separated almost or completely by baseline indicating the potential of IM-MS for separating oligosaccharides derived from glycoproteins [4]. They then demonstrated the feasibility of IM-MS for separating simple carbohydrates in a mixture by running the sample mixture of three trisaccharides through IM-MS and showing that they were baseline separated (see Figure 5) [4]. By combining experimental results and the calculation of resolving power (Equation (3)), they concluded that IM-MS was powerful enough to separate simple carbohydrate mixtures unambiguously for the first time [4]. Based on the resolving power of APIMS-MS as a separation tool and the higher stability of sodium adducts of carbohydrates in the gas phase [4,66], Hill and coworkers used APIMS-MS to separate anomeric methyl glycosides via their sodium adducts. Free reducing sugars, as metal ion adducts, were also resolved into more than one peak, possibly representing different anomeric configurations or ring forms of the reducing sugars [12]. Their predictions that: (a) using APIMS-MS to separate larger oligosaccharides in mixtures considering specific reducing sugars had unique mobility profiles, (b) optimizing instrumental parameters through the alternations of metal ions adducted and drift gases that applied to the carbohydrate structural analysis system, were later confirmed by other research groups with more details [12].

The performance of IM-MS for characterization of carbohydrates can be improved by analyzing alkali metal ion adducts. For example, Uhrin and coworkers demonstrated the advantages of analyzing sodium adducts of carbohydrates for structural analysis [66]. This group conducted conformational analyses on oligosaccharides derived from heparin through APIMS-MS followed by molecular modelling. They demonstrated the importance of sodium ions stabilizing carbohydrate ions in the gas phase by comparing the experimental CCSs obtained in the presence of sodium ions in the system with theoretical CCSs obtained by modelled molecules in the absence of sodium ions [66]. Fenn and McLean developed a CCS database of over 300 carbohydrates and demonstrated the improved resolving power of IM-MS after derivatizing carbohydrate samples with alkali metal ions prior to mass analysis [67]. They showed that different alkali metals for metal-coordination can be used to alter the CCSs obtained [67]. Their study was the first one to calculate CCSs and CCSs of in-source decay fragments of large sets of carbohydrate standards [67]. The impact of the addition of sodium ions on drift times of structural isomers of monosaccharide methyl glycosides was studied by Hill and coworkers. Sodium ions were shown to affect drift times depending on the coordination strength, configuration and coordination geometry of -OH and -OCH_3_ groups, and the coordination geometry induced by Na^+^ adduction [15]. Later, they provided direct evidence for the isomeric heterogeneity of carbohydrate product ions for the first time, demonstrated the ability of IM-MS to discriminate the isomeric heterogeneity of oligosaccharide samples without prior knowledge, underlined the importance of rapid resolution prior to mass analysis for the structure and the importance of utilizing fragmentation patterns for individual identification of oligosaccharide isomers that might have contributed to isomeric mobility peaks [32,63].

IMS can be used in conjunction with fragmentation studies to provide further carbohydrate structural information. Fenn and McLean’s study on CCSs of in-source decay fragments demonstrated the possibilities to add fragmentation studies into the analytical system for higher sensitivity and more complete resolution of specific carbohydrates, added either prior to IMS or prior to MS [67]. Seven isobaric disaccharides selected in Clemmer’s study were all different in linkage types or configurations and were separated through IMS followed by collision-induced dissociation (CID) or vacuum ultraviolet photodissociation (VUVPD) and tandem MS or MS*n* [37]. Both linkage and configurational dissociation patterns were observed in CID and VUVPD, but more complex fragmentation patterns were only observed in experiments with VUVPD [37]. With the production of unique photofragment ions, VUVPD has the potential to distinguish disaccharide isomers from mixtures containing simple carbohydrates [37].

In addition to utilizing different metal ions to coordinate with carbohydrates and using different drift gases with different properties, efforts have also been made to improve IM-MS through the derivatization of samples in other ways or through other additional instruments. For instance, the selectivity of IM-MS could be improved by derivatizing carbohydrate ions with boronic acids for more structural information [64]. In this study, a set of carbohydrate ions, from disaccharides to pentasaccharides, were derivatized by two distinct boronic acids, phenylboronic acid (PBA) or ferrocene boronic acid (FBA), and CCSs of the carbohydrate ions were calculated to compare with non-derivatized ones [64]. Although the effects of the two boronic acids on CCSs of derivatized carbohydrates were different, the desired conformational mobility shift was achieved for most samples and it turned out that derivatized carbohydrates were more advantageous than non-derivatized ones in three main aspects: (a) adjustability of isobar separation; (b) improvement in separation efficiency; and (c) possibility to serve as fragment labels for tandem MS studies [64].

The utilization of additional techniques with IM-MS can also improve its resolving power. Hill and coworkers provided an example by combining APIMS-MS with HPLC and tandem MS: HPLC-APIMS-ion trap MS*n*. APIMS–ion trap MS*n* was capable of discriminating oligosaccharide isomers in a mixture and providing dissociation spectra for each isomeric ion on the millisecond timescale without any prior assumptions or knowledge about their structures [13]. With the development of DTIMS and TWIMS, the resolving power has been greatly improved [11]. Scrivens and Bowers and coworkers compared the two techniques for separating sodiated isomeric penta- and hexasaccharides [11]. They found that TWIMS was more likely to be used as an analytical tool for screening complex glycans or other biological samples, although the resolving power of DTIMS was slightly higher than TWIMS [11]. However, the details of the comparison were not clear until the study conducted by Hill’s group. Hill and coworkers conducted identical studies in both IMS systems to make a direct and detailed comparison between them through small, relatively rigid isomers and the results were in accordance with those of Scrivens and Bowers [15,63]. By separating a complete series of structural isomers of monosaccharide methyl glycosides for the first time, Hill’s group revealed systematic structural information associated with the mobility of isomers through the calculations of *K*_0_ and *Ω* [15]. In another study, a hybrid IM-MS instrument, (IM-MS)^2^, was described, which was the first instrument that combined DTIMS and TWIMS, mass selection and mobility selection, with tandem MS [63]. With more information for the characterization of carbohydrate isomers that could be not readily obtained through traditional CID, the hybrid instrument provided a novel method to evaluate the isomeric heterogeneity of product ions through mass and mobility selected precursor ions, and to unambiguously assign carbohydrate isomers in mixtures [63]. Moreover, the possibility to calibrate data obtained by TWIMS through additional DTIMS was first described in Hill’s report [63]. Another method for calibrating TWIMS was reported by Pagel [19,68]. Pagel constructed a database for the CCS calibration of TWIMS utilizing a set of positively charged sodiated N-glycans that were obtained from commercially available glycoproteins [19]. He subsequently presented a database for the CCS calibration of TWIMS with over 500 reference CCSs for negatively charged N-glycans and their fragments [68]. Although the impact of different drift gases on general separation among biological classes was small, one still needed to consider taking calibration methods when utilizing CCS values derived from the other drift gases since nitrogen CCS values were systematically larger than helium values [57]. Yamaguchi utilized HILIC, a pre-separation tool, to couple with a traditional IM-MS instrument and showed that this new technique could facilitate the identification of glycans and the construction of databases [56]. Pagel introduced an energy-resolved ion mobility-mass spectrometer, utilizing the different energies when fragments were activated in the gas phase via CID, to improve the resolving power of traditional IM-MS instruments [65].

With the enhanced resolving power of IM-MS, Seeberger and Pagel showed that it could separate coexisting carbohydrate anomers even when the relative concentration of the minor anomer was as low as 0.1% [14]. Hill’s group discriminated carbohydrate isomers with negative ion mode of TWIMS and defined more detailed criteria for databases with appropriate carbohydrate standards [16].

### 4.2. Complex carbohydrates

IM-MS can also be utilized to detect and analyse protein glycosylation. Glycosylation is the most common co- and post-translational modification of proteins with numerous functions and it is responsible for physicochemical properties and conformational stability of proteins, protein folding, membrane attachment, cell-to-cell interactions and transportation [11,36]. Glycosylation is not a template-mediated modification and it thus produces heterogeneous structures. Therefore, much effort has been expended in searching for instruments with high speed, high sensitivity and high resolution such as IM-MS [35,36]. The use of IMS prior to MS greatly aids the structural determination of N-glycans, especially at low concentrations or in contaminated samples. This is because ion extraction of different charge states by IMS could reduce contamination greatly and therefore reveal the presence of minor components from the sample that were masked by other major components in different charge states [69]. Considering the presence and/or abundance of specific glycans as biomarkers that change with disease progression [1], much biomedical research is aimed at determining the glycan profile. The main impediment to this research has been the complexity of mixtures derived from biological sources [70]. Clemmer and coworkers examined N-glycans isolated from ovalbumin by IM-MS and compared the experimental CCSs of glycan isomers with theoretical CCSs obtained from molecular modelling to deduce features of the isomers [36]. They showed that IM-MS was a promising technique for assigning structural isomers, and predicted the future development of multidimensional IM-MS techniques for the detection of glycan heterogeneity and glycan isomers [36]. Clemmer’s group was also the first to utilize IM-MS and PCA to analyse glycans and glycoproteins related to liver cancer and cirrhosis, while previous studies focused only on the comparison of glycans between healthy and diseased tissue with less sensitive instrument such as ESI-MS and MALDI-MS [35]. In this study, they demonstrated the feasibility of IM-MS to probe disease states, because abnormal ion mobility distributions of isomers that indicate glycan conformational and isomeric composition could be used as markers for disease state [35]. Vakhrushev et al. demonstrated the reliability of this technique and explored its clinical applications [24]. In this study, both N- and O-glycans from urine samples of patients with congenital disorders of glycosylation (CDG) were separated by IM-MS/MS [24]. With additional CID prior to mass analysis, the separation of overlapping regions in the MS spectra were further enhanced, providing evidence of the utility of this technique for mapping carbohydrate epitopes involved in CDG, and in determining the extent of sialylation and fucosylation in cinical samples [24]. The discrimination of epimeric glycans by IM-MS has also been demonstrated, suggesting this technique may be useful for carbohydrate sequencing [71]. In another study, IM-MS analysis was used to successfully characterize and separate individual heparin octasaccharide isomers with different degrees of sulfation and to subsequently measure their conformations [26]. Based on previous studies, Pagel’s group showed that glycan isomers could be differentiated by their drift times as well as their absolute CCS values and they emphasized the potential to utilize derived CCSs information as a database searching parameter, for a more direct understanding of glycan structures [19]. With the maturity of IM-MS applications in glycans, it was then used to observe glycans from more complex sources. For example, Crispin et al. provided evidence of IM-MS being a suitable analytical tool for identifying the composition of glycans from Semliki Forest virus (SFV), a prototypic alphavirus [72]. Rashid et al. demonstrated the ability of IM-MS, in a negatively charged mode, to identify a branching enzyme from a complex maltooligosaccharide mixture [73]. With the popularity of IM-MS, databases of glycoconjugate CCSs with both DTIMS and TWIMS in helium and nitrogen are under construction [27]. Later, the combination of IM-MS with chromatographic separations was further combined with negative mode CID, the novel coupling technique was more capable of providing rapid and reliable structural information of N-glycans than other techniques available [70]. Moreover, it was shown that IM-MS is a powerful tool for determining the impact of reduction, a method to avoid the presence of multiple peaks from α- and β-anomers, on the production of diagnostic fragment ions and on the ion mobility properties of N-glycans [74].

IM-MS is also powerful for analysing more complex mixtures derived from abnormal biological phenomena. For example, cooperative binding of concanavalin A tetramer to a set of polysaccharide ligands was examined by the combined collision-induced unfolding (CIU) and traditional IM-MS for a better understanding of its unfolding properties [50]. Pritchard et al. utilized IM-MS coupled with HPLC to characterize glycan structures shown on native HIV-1 envelope for the design of an HIV vaccine [25]. Zhao et al. emphasized the advantages of TWIMS for investigating conformational changes of protein induced by the binding of glycosaminoglycan ligands through the system of Antithrombin III (ATIII) and Arixtra (fondaparinux) [28]. More recently a study regarding isomeric chondroitin sulfate disaccharides was conducted with the utilization of two methods together: IM-MS and pulsed-field gradient (PFG) NMR. It was shown that without the absolute need for standards, IM-MS and (PFG) NMR was capable of characterizing carbohydrate isomers in mixtures [75].

### 4.3. Oligonucleotides

IM-MS is also utilized to analyse compounds that contain saccharides, such as oligonucleotides. Bowers was the earliest to study gas phase conformations of dinucleotides utilizing IM-MS and to propose folding energetics through systematic annealing and minimizations [76]. Based on Bowers’ work and the prior literature regarding the utilities of IM-MS on peptides, Koomen et al. demonstrated the potential of IM-MS to separate and characterize native and derivatized oligonucleotides of different lengths, to resolve platinum modified DNA from the corresponding native oligonucleotides and to achieve oligonucleotide sequencing [77]. McLean’s study focused on resolution of IM-MS, and proved that IM-MS could baseline separate two oligonucleotides with different orders of four identical bases (i.e., CGAT and TGCA) [34].

## 5. Conclusions

IM-MS is a promising separation tool for applications in many fields within the biosciences, with a growing number of applications in carbohydrate chemistry and glycobiology. The main reasons IM-MS is so powerful for biological analyses are: (a) IM-MS only requires a millisecond timescale to analyse sample mixtures, facilitating the detection of biological samples that might change over time; (b) its high sensitivity benefits the detection of molecules with extremely low concentrations in biological processes; and (c) its high resolving power allows the differentiation of similar molecular structures. However, IM-MS is still not powerful enough to separate structures that are almost identical. Efforts have been made to hybridize IM-MS with different kinds of IMS and MS or to combine IM-MS with chromatographic separations, with fragmentation tools, and most recently, with NMR to fill the gaps in isomer structural elucidation between the gas phase and solution phase. In the future, the use of IM-MS in the glycosciences should continue to expand and might be utilized in research on disease progression, medicine and vaccine design, to name a few.
